# The molecular epidemiology and clinical implication of methicillin-resistant *Staphylococcus aureus* (MRSA) sequence types in pediatric bacteremia: a restrospective observational study, 2016–2021

**DOI:** 10.1186/s12879-023-08914-5

**Published:** 2024-02-24

**Authors:** Gahee Kim, Sanghoon Lee, Yonghee Lee, Jung Hwa Kim, Jina Lee

**Affiliations:** 1 Department of Pediatrics, Asan Medical Center, University of Ulsan College of Medicine, Seoul, Republic of Korea; 2 Department of Pediatrics, Chosun University hospital, Gwangju, Republic of Korea; 3 Department of Pediatrics, Gangwon National University hospital, Chunchen, Gangwon-do Republic of Korea

**Keywords:** MRSA, ST72, ST5, ST1, Clinical severity, MLST, Children

## Abstract

**Background:**

While there is a high burden of methicillin-resistant *Staphylococcus aureus* (MRSA) infections among pediatric patients, studies on the molecular epidemiology of MRSA infections in Korean children since the 2010s are lacking. This study aimed to investigate the molecular genotypes and clinical characteristics of MRSA isolates from children with MRSA bacteremia at Asan Medical Center Children’s Hospital from 2016 to 2021.

**Methods:**

Clinical data were retrospectively reviewed, and the molecular types of MRSA were determined using multilocus sequence typing (MLST) and Staphylococcal cassette chromosome mec (SCCmec) typing.

**Results:**

The overall methicillin resistance rate of *S. aureus* bacteremia was 44.8% (77/172); 49.5% in the period 2016–2018 (period 1) and 37.3% in the period 2019–2021 (period 2) (*P* = 0.116). Community-acquired infections accounted for only 3.9% of cases. The predominant ST group was ST72 group (67.6%), followed by ST5 group (18.9%) and ST1 group (5.4%). The proportion of ST5 was significantly lower in period 2 compared to period 1 (*P* = 0.02). Compared to the ST5 and ST1 groups, the ST72 group exhibited lower overall antibiotic resistance and multidrug-resistant (MDR) rates (12.0% [6/50] in ST72 group vs. 100.0% [14/14] in ST5 group vs. 50.0% [2/4] in ST1 group; *P* < 0.001). In the multivariate analysis, the ST1 group was an independent risk factor for 30-day all-cause mortality (aOR, 44.12; 95% CI, 3.46–562.19).

**Conclusion:**

The ST72-MRSA strain remained the most frequently isolated genotype in Korean children, while the ST1 group emerged as an independent risk factor for 30-day all-cause mortality in pediatric MRSA bacteremia. Ongoing efforts to uncover the evolving epidemiology of MRSA are essential for developing effective strategies for prevention and treatment.

**Supplementary Information:**

The online version contains supplementary material available at 10.1186/s12879-023-08914-5.

## Introduction

*Staphylococcus aureus* bacteremia (SAB) can lead to substantial morbidity and mortality, even with appropriate antibiotic treatment [1]. The incidence of *S. aureus* bacteremia varies among pediatric patients, ranging from 3.7 to 15 per 100,000 individuals in different regions, with particularly high rates among infants under the age of one-year [1–3]. MRSA bacteremia has been associated with higher mortality rates compared to methicillin-susceptible *S. aureus* (MSSA) bacteremia [4]. In South Korea, the prevalence of methicillin-resistant *S. aureus* (MRSA) among pediatric patients with SAB admitted to a tertiary hospital was notably elevated, ranging from 65.2 to 76.1% between 2002 and 2016 [5].

Understanding the evolving molecular epidemiology of MRSA is important to effectively control MRSA transmission. Molecular typing methods, such as multilocus sequence typing (MLST) and Staphylococcal cassette chromosome mec (SCCmec) typing, allow the investigation of genetic backgrounds and spread of MRSA isolates across different time periods and geographic regions [6]. In South Korea, the sequence type (ST)72-MRSA has been the predominant genotype in both community and hospital settings since the mid-2000s, not only among adults but also in children [7, 8]. However, there is a lack of studies on the changes in the molecular epidemiology of MRSA infections among Korean children since the 2010s.

Moreover, the impact of specific MRSA genotypes on clinical outcomes remains incompletely understood. While there have been reports focusing on ST8-MRSA (USA300), that have not yielded definitive conclusions [9–11]. For instance, ST8-MRSA has been associated with severe sepsis in otherwise healthy children [10] and higher mortality in bacteremic patients [9]. However, another study has suggested that ST8 itself may not be directly linked to persistent bacteremia or treatment failure [11].

We aimed to investigate the molecular types and clinical characteristics of MRSA isolates obtained from children with bacteremia admitted to a tertiary children’s hospital in Korea over a span of 6 years. We also aimed to assess the potential impact of MRSA STs on antibiotic resistance patterns, clinical features, and overall outcomes.

## Materials and methods

### Study population and definitions

This retrospective observational study focused on pediatric patients aged ≤ 19 years who had MRSA bacteremia at Asan Medical Center Children’s Hospital, a 270-bed university-affiliated tertiary center located in Seoul, Korea, between January 2016 and December 2021. Relevant demographic and clinical data, including underlying diseases, primary sources of infection, severe presentation, persistent bacteremia, and mortality, were extracted from electronic medical records. Only the initial MRSA isolates detected during a single episode of MRSA bacteremia were included in the analysis with duplicates from the same patient being excluded. Although at least two sets of blood cultures were drawn for diagnosis in most cases of *S. aureus* bacteremia, the detection of *S. aureus* from blood cultures is considered a true infection due to the high probability of its true positivity of *S. aureus* from blood cultures and the clinical significance of *S. aureus* bacteremia.

MRSA infections were classified as community-onset (CO) if the first positive blood culture was obtained in an outpatient setting or within 48 h of hospital admission, and as hospital-onset (HO) infections if the first positive culture was obtained ≥ 48 h after hospital admission. Further classification of CO infections included community-acquired (CA) or healthcare-associated (HCA) infections based on healthcare-associated risk factors defined by Friedman et al. [12].

Primary bacteremia is defined as a condition where bacteremia occurs without an apparent or localized source of infection. The primary source of bacteremia was categorized as central line-associated bloodstream infection (CLABSI), bone and joint infection (BJI), skin soft tissue infection (SSTI), surgical site infection (SSI), or pneumonia, as per the definitions provided by the Centers for Disease Control and Prevention (CDC)’s National Healthcare Safety Network (NHSN) [13]. Infective endocarditis was defined according to the modified Duke criteria [14]. Severe presentation was defined as an episode requiring the initiation of vasopressors, an escalation in their dosage, or the commencement of mechanical ventilation within 48 h of MRSA bacteremia onset. Separate episodes were considered for cases in which MRSA was isolated again from the blood after at least a 3-month interval of culture-negativity. Persistent bacteremia at day 3 and day 7 was defined as a positive blood culture for more than 3 or 7 days, respectively, despite appropriate anti-MRSA therapy initiation. Mortality was evaluated by 30-day all-cause mortality and MRSA-related mortality, which was attributed directly to MRSA bacteremia without any other reasonable explanation. Clinical and microbiological outcomes were evaluated based on 30-day all-cause mortality and persistent bacteremia at day 7, respectively.

### Strain identification and antimicrobial susceptibility testing

Strain identification and antimicrobial susceptibility testing were carried out using MicroScan WalkAway 96-Combo Pos 28 panels (Siemens, West Sacramento, CA, USA) for *S. aureus* isolates. The panel contained six wells with vancomycin concentrations ranging from 0.5 to 16.0 µg/mL. Additionally, the panel encompassed various antibiotics for susceptibility testing, including oxacillin, penicillin, ampicillin, amoxicillin/clavulanate, azithromycin, clindamycin, ciprofloxacin, daptomycin, erythromycin, fusidic acid, fosfomycin, gentamycin, imipenem, levofloxacin, linezolid, mupirocin, moxifloxacin, rifampin, quinupristin/dalfopristin, trimethoprim/sulfamethoxazole (TMP/SMX), tetracycline, and teicoplanin.

MRSA isolates of intermediate resistance or full resistance were defined as resistant. The in vitro macrolide-lincosamide-streptogramin _B_ (MLS_B_)-inducible phenotype was detected by the D-zone test (double-disk diffusion test). Multidrug resistance (MDR) was defined as acquired non-susceptibility to at least one agent in three or more different classes of non-ß-lactam antibiotics [15]. The minimal inhibitory concentration (MIC) breakpoints were interpreted according to the standard criteria of the Clinical and Laboratory Standards Institute (CLSI) document M100 [16].

### Multilocus sequence type (MLST) analysis and SCC ***mec*** typing

MLST analysis and SCC*mec* typing were conducted using the first MRSA isolates obtained during each bacteremic episode and stored at -70℃ for molecular analysis. MLST was performed by polymerase chain reaction (PCR) amplification and sequencing of seven housekeeping genes (*arc*, *aroE*, *glpF, gmk, pta, tpi, ygiL*), as described previously [17]. Each sequence was submitted to the MLST database (http://pubmlst.org) to assign an allelic profile and ST. Staphylococcal cassette chromosome *mec* (SCC*mec*) typing was carried out using multiplex PCR, as described previously [18].

### Statistical analyses

The incidence of SAB was calculated as the annual number of episodes per 1,000 inpatients. The analysis of SAB incidence was performed using a Poisson regression model. For categorical variables, Chi-square or Fisher’s exact test was used, while the Mann–Whitney U test was employed for continuous variables. These tests were utilized to compare data between two periods (period 1, 2016–2018, and period 2, 2019–2021) and among three distinct ST groups: ST72, ST5, and ST1, each with its corresponding specific single locus variants(SLVs).

Univariate and multivariate logistic regression analyses were performed to identify independent risk factors associated with persistent bacteremia lasting more than 7 days and 30-day all-cause mortality. The multivariable analysis included variables with a *P*-value < 0.20 in univariate analysis. Statistical analysises were performed using SPSS version 23.0 (SPP Inc., Chicago, IL, USA). All significance tests were two-tailed, and *P*-values of ≤ 0.05 were considered statistically significant.

## Results

### Study population

**Fig. 1:**
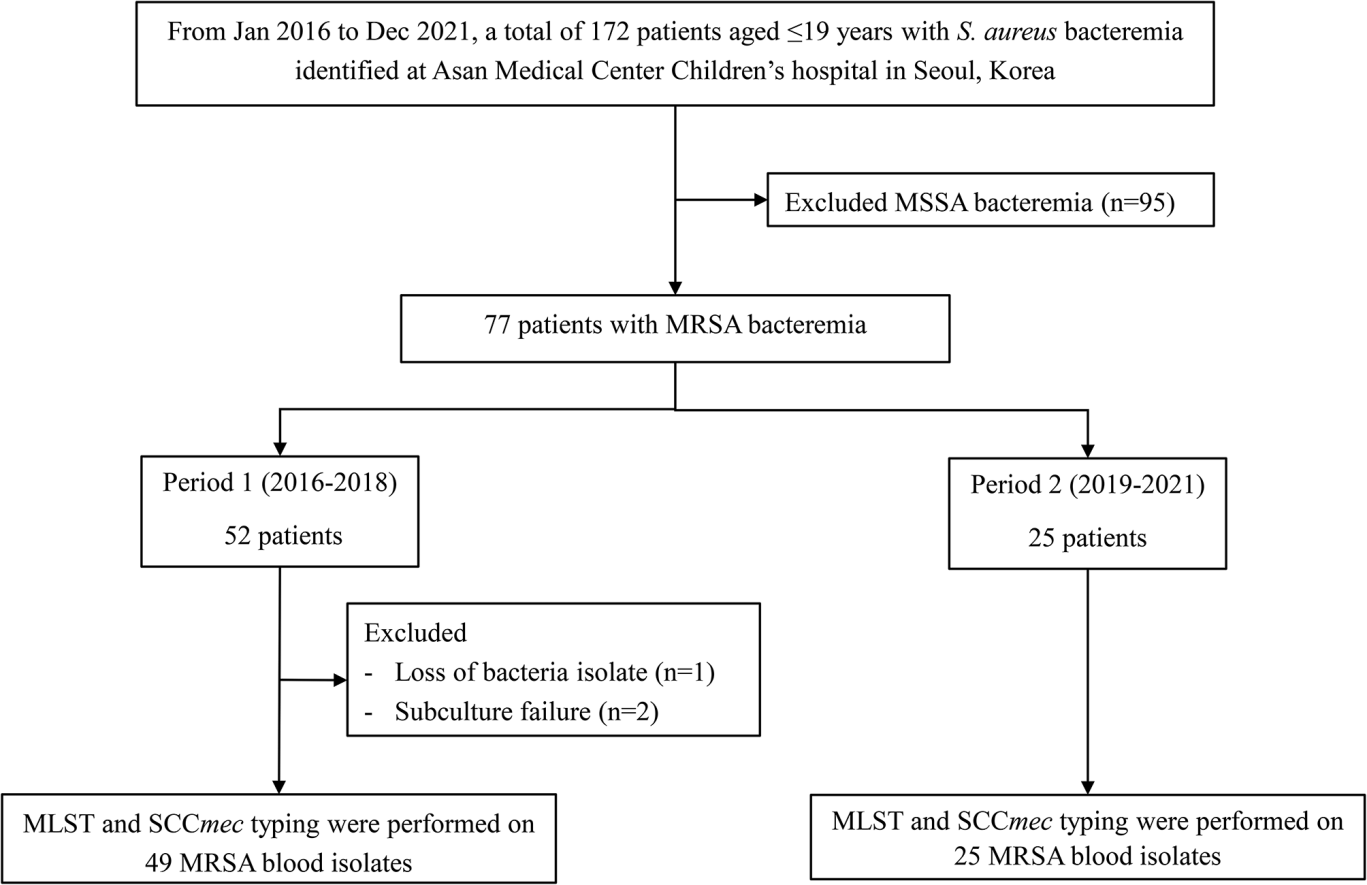
Changes in antimicrobial resistance rate among MRSA blood isolates (total n = 77). MLST, multilocus sequence typing; SCCmec, Staphylococcal cassette chromosome mec

**Fig. 2:**
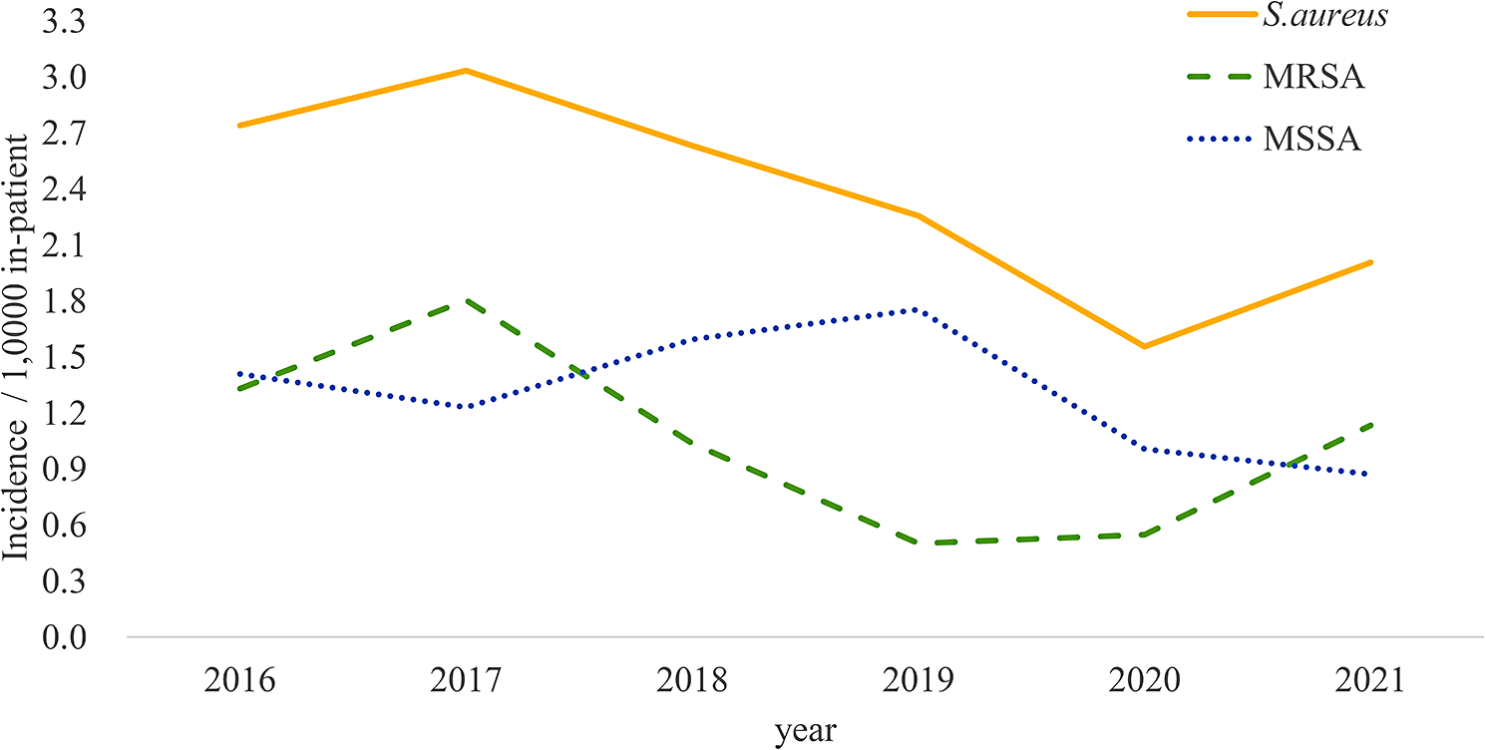
Annual incidence of *Staphylococcus aureus* bacteremia per 1,000 patients during 2016–2021

During the study period from January 2016 to December 2021, a total of 172 patients encountered SAB, resulting in an overall incidence rate of 2.39 per 1,000 patient-years (Fig. 1). An evident annual decline of 14% was observed in the overall incidence of SAB (from 2.74/1,000 inpatients in 2016 to 2.01/1,000 inpatients in 2021; *P* = 0.002) (Fig. 2). This reduction was primarily attributed to a yearly decrease of 23% in the incidence of MRSA bacteremia (from 1.33/1,000 inpatients in 2016 to 1.13/1,000 inpatients in 2021; *P* = 0.001). The overall methicillin resistance rate among SAB cases was 44.8% (77/172); this was 49.5% (52/95) during the period 2016–2018 (period 1) and 37.3% (25/67) during the period 2019–2021 (period 2) (*P* = 0.116).

The demographic and clinical characteristics of the 77 patients with MRSA bacteremia are presented in Table 1. The median age of the patients was 1.0 year (interquartile range [IQR], 0.0–6.0). 71 (92.5%) had underlying medical conditions, with 19 having hemato-oncologic disease and 11 having a history of stem cell or solid organ transplantation. Fifty-five cases (71.4%) were classified as HO MRSA infections, with 42 (54.6%) of them occurring during stays in intensive care units (ICUs): 16 in the neonatal ICU (NICU) and 26 in the pediatric ICU (PICU). During this study period, routine nasal surveillance for MRSA was conducted for all patients in the PICUs upon admission to PICU and then weekly thereafter. Pior to the onset of MRSA bacteremia, 43 (55.8%) patients were colonized with MRSA in the anterior nares. The most common primary source of MRSA bacteremia was the CLABSI (50.6%), followed by SSTI (10.4%). Primary bacteremia accounted for 19.5% of all infections. Out of the 39 MRSA CLABSI episodes, 10 (25.6%) were complicated bacteremia, including venous thrombi (n = 6), septic emboli (n = 3), and infective endocarditis (n = 1). The four instances of SSI involved deep-seated infections, including mediastinitis (n = 2) and complicated pleural effusion (n = 2). Only 3.9% of MRSA bacteremia occurred in previously healthy children without healthcare risk factors, with SSTI (n = 2) and BJI (n = 1) as the primary foci. All patients with MRSA bacteremia were treated with in vitro susceptible glycopeptides within 48 h of bacteremia onset.

**Table 1:** Demographic and clinical characteristics of pediatric MRSA bacteremia between the two study periods

Characteristics	Total (n = 77)	Period 1 (2016–2018) (n = 52)	Period 2 (2019–2021) (n = 25)	*P-*value
Age, year (median, IQR)	1.0 (0.0–6.0)	0.0 (0.0–3.0)	2.0 (0.0–9.0)	0.09
Male	45(58.4)	31 (59.6)	11 (40.4)	0.76
Underlying disease	71 (92.5)	47 (90.4)	21 (96.0)	0.66
Heart disease	26 (33.8)	17 (32.7)	9 (36.0)	0.77
Gastrointestinal disease	19 (24.7)	11 (21.2)	8 (32.0)	0.30
Hemato-oncologic disease	19 (24.7)	13 (25.0)	6 (24.0)	0.92
Neurologic disease	12 (15.6)	3 (5.8)	9 (36.0)	< 0.01
Renal disease	7 (9.1)	4 (7.7)	3 (12.0)	0.68
Chronic lung disease	7 (9.1)	4 (7.7)	3 (12.0)	0.68
Endocrinologic disease	7 (9.1)	4 (7.7)	3 (12.0)	0.68
Metabolic disease	5 (6.5)	3 (5.8)	2 (8.0)	0.66
Preterm birth (GA < 37weeks)	14 (18.2)	11 (21.2)	3 (12.0)	0.53
Stem cell transplantation ^a^	7 (9.1)	5 (9.6)	2 (8.0)	> 0.99
Solid organ transplantation ^a^	2 (2.6)	1 (1.9)	1 (4.0)	0.55
Recent surgery within 1 year	46 (59.7)	28 (53.8)	18 (72.0)	0.13
Immunosuppressive treatment ^b^ within 1 month	19 (25.0)	12 (23.1)	7 (29.2)	0.57
Presence of central venous catheter	65 (84.4)	42 (80.8)	23 (92.0)	0.32
ICU stay at the onset of bacteremia	42 (54.5)	30 (57.7)	12 (48.0)	0.42
MRSA colonization before infection	43 (55.8)	30 (57.7)	13 (52.0)	0.64
Place of acquisition				
Hospital onset				
Hospital-acquired infection	55 (71.4)	37 (71.2)	18 (72.0)	0.94
Community onset				
Healthcare-associated infection	19 (24.7)	14 (26.9)	5 (20.0)	0.51
Community-acquired infection	3 (3.9)	1 (1.9)	2 (8.0)	0.25
Primary source of infection				
Central venous catheter infection ^c^	39 (50.6)	23 (44.2)	16 (64.0)	0.10
Skin/soft tissue infection	8 (10.4)	6 (11.5)	2 (8.0)	> 0.99
Pneumonia	6 (7.8)	4 (7.7)	2 (8.0)	> 0.99
Surgical site infection ^d^	4 (5.2)	3 (5.8)	1 (4.0)	> 0.99
Infective endocarditis	3 (3.9)	2 (3.8)	1 (4.0)	> 0.99
Bone and joint infection	2 (2.6)	0 (0.0)	2 (8.0)	0.10
Primary bacteremia	15 (19.5)	14 (26.9)	1 (4.0)	0.03
Severe presentation	13 (16.9)	7 (13.5)	6 (24.0)	0.33
Vasopressor use	8 (10.4)	6 (11.5)	2 (8.0)	> 0.99
Mechanical ventilation	7 (9.1)	3 (5.8)	4 (16.0)	0.26
Recurrence of MRSA bacteremia	7 (9.1)	4 (7.7)	3 (12.0)	0.68
Duration of bacteremia, days (median, IQR)	3.0, (1.0–4.0)	2.0, (1.0–3.0)	3.0, (1.0–7.0)	0.30
Persistent bacteremia				
3-day	22 (28.9)	11 (21.6)	11 (44.0)	0.04
7-day	9 (12.0)	4 (7.8)	5 (20.8)	0.13
Mortality				
MRSA-related mortality	3 (3.9)	3 (5.8)	0 (0.0)	0.55
30-day all-cause mortality	8 (10.4)	5 (9.6)	3 (12.0)	0.71

Data are presented as number (%) unless otherwise specified

Abbreviations: IQR, interquartile range; ICU, invasive care unit; GA, gestational age

Notes: ^a^ Solid organ transplantation (SOT) or stem cell transplantation (SCT) received within 1 year. Both episodes in SOT recipients occurred within 3 months after the SOT, while all seven episodes in SCT recipients occurred within 1 year post-SCT.

^b^ Immunosuppressive treatment includes high-dose steroids (≥ 14days), and immunosuppressant use (≥ 7 days) within 1 month of bacteremia onset

^c^ Among these, 9 (23.1%) had complicated central venous catheter-related infections such as venous thrombi

^d^ Two cases had mediastinitis, and the other two cases had complicated pleural effusion

Severe presentation, requiring vasopressors administration or mechanical ventilation within 48 h of bacteremia onset, was observed in 13 cases (16.9%), and the 30-day all-cause mortality rate was 10.4% (8/77). Among the three MRSA-related deaths, the median time from bacteremia onset to death was 5.0 days (IQR, 1.0–10.5). The median duration of bacteremia was 3.0 days (IQR, 1.0–4.0), with 22 (28.9%) patients experiencing persistent bacteremia at day 3 and 9 (12.0%) at day 7, despite initiation of appropriate antibiotic therapy.

Comparing period 2 to period 1, the rate of primary bacteremia was significantly lower (26.9% [14/52] in period 1 vs. 4.0% [1/25] in period 2; *P* = 0.03), and persistent bacteremia at day 3 was observed more frequently (21.6% [11/52] in period 1 vs. 44.0% [11/25] in period 2; *P* = 0.04).

### Molecular characterization

A total of 74 available MRSA isolates underwent molecular characterization, all of which tested positive for the *mec* A gene. Among these, SCC*mec* types I, II, and IV were detected, with SCC*mec* type IV being the most prevalent (71.4%). The proportion of SCC*mec* type IV increased during period 2 (84.0%) compared to period 1 (71.4%) (*p* = 0.2) (Table 2). Within the ST72 group (n = 50), which included ST72 (n = 47) and its single locus variants (SLVs) ST2084 (n = 2) and a novel SLV (n = 1), SCC*mec* type IV was most frequently detected (n = 47; 94.0%), with three isolates exhibiting SCC*mec* type II (n = 2) and SCC*mec* type I (n = 1). All ST5 isolates (n = 9) and its SLVs, including ST632 (n = 1) and novel SLVs (n = 3) (referred to hereafter as the ST5 group), harbored only SCC*mec* type II. Among the ST1 group including ST1 (n = 3) and one novel SLV of ST1 (n = 1), only SCC*mec* type IV was identified.

**Table 2:** Molecular characteristics of MRSA blood isolates according to the epidemiologic settings across the study periods (n = 74)

	Period 1 (2016–2018) (N = 49)	Period 2 (2019–2021) (N = 25)	*P-*value	Hospital-onset	Community-onset		*P-*value
				(N = 52)	Healthcare-associated (N = 19)	Community-acquired (N = 3)	
**SCC** ***me*** **c type**							
SCC*me*c I (n = 2)	1 (2.0)	1 (1.0)	> 0.99	1 (1.9)	1 (5.3)	0 (0.0)	0.509
SCC*me*c II (n = 16)	13 (26.5)	3 (12.0)	0.151	14 (26.9)	2 (10.5)	0 (0.0)	0.125
SCC*me*c IV (n = 56)	35 (71.4)	21 (84.0)	0.233	37 (71.2)	16 (84.2)	3 (100.0)	0.238
**ST groups**							
ST1 group ^a^ (n = 4)	3 (6.1)	1 (4.0)	> 0.99	4 (7.7)	0 (0.0)	0 (0.0)	0.311
ST5 group ^b^ (n = 14)	11 (22.4)	3 (12.0)	0.358	13 (25.0)	1 (5.3)	0 (0.0)	0.052
ST72 group ^c^ (n = 50)	31 (63.3)	19 (76.0)	0.268	31 (59.6)	17 (89.5)	2 (66.7)	0.031
Others (n = 6)	4 (8.2) ^d^	2 (8.0) ^e^	> 0.99	4 (7.7) ^f^	1 (5.3) ^g^	1 (33.3) ^h^	> 0.99

Data are presented as numbers (%) of patients

Abbreviations: SCCmec, Staphylococcal cassette chromosome mec; ST, sequence type; SVLs, single locus variants

Notes: ^a^ ST1 (n = 3) and its SLV (n = 1), including a novel SLV (n = 1)

^b^ ST5 (n = 9) and its SLVs (n = 5), including ST632 (n = 1) and novel SLV (n = 4). A total of nine ST5 MRSA strains were identified only during period 1 (18.4% [9/20] in period 1 vs. 0% [0/25] in period 2, *p* = 0.02)

^c^ ST72 (n = 47) and its single locus variants (SLVs) (n = 3), including ST2084 (n = 2) and a novel SLV (n = 1)

^d^ Other STs in period 1: ST22 (n = 2), ST30 (n = 1), ST89 (n = 1), and ST1181 (ST8 SLV) (n = 1)

^e^ Other STs in period 2: ST8 (n = 1), ST 188 (n = 1)

^f^ Other STs of hospital-onset MRSA: ST22 (n = 2), ST30 (n = 1), ST188 (n = 1), and ST1181 (ST8 SLV) (n = 1)

^g^ indicates ST89

^h^ indicates ST8. This single ST8 MRSA isolate with SCC*mec* type IV was isolated from a community-acquired infection, involving a retropharyngeal abscess extending into the mediastinum

In period 2 compared to period 1, the proportion of the ST72 group was higher (63.3% [31/49] vs. 76.0% [19/25]; *P* = 0.27), and that of the ST5 group was lower (22.4% [11/49] vs. 12.0% [3/25]; *P* = 0.36), although there was no statistical significance. Furthermore, all the ST5 MRSA strains were identified only during period 1 (18.4% [9/20] in period 1 vs. 0% [0/25] in period 2; *P* = 0.02).

Regardless of hospital or community settings, ST72 group predominated (67.6%), followed by ST5 group (18.9%) and ST1 group (5.4%). Notably, ST5 and ST1 were exclusively observed in HO infections. The ST72 group showed a lower likelihood of being associated with HO infection (62.0% [31/50] vs. 94.4% [17/18]; *P* = 0.014) and previous MRSA colonization (46.0% [23/50] vs. 88.9% [16/18], *P* = 0.002) compared to the ST1 or ST5 groups.

### Clinical significance of three different ST groups

Regarding clinical outcomes, the 30-day all-cause mortality rates for each ST group were as follows: 75% (3/4) for the ST1 group, 10% (5/50) for the ST72 group, and none for the ST5 group. After adjusting for primary bacteremia and ST1 group, multivariate analysis revealed that the ST1 group was an independent risk factor for 30-day all-cause mortality (adjusted odds ratio [aOR], 44.12; 95% confidence interval [CI], 3.46–562.19) (Table 3).

**Table 3:** Risk Factors for clinical and microbiological outcomes in pediatric MRSA bacteremia

	30-day all-cause mortality (n = 8)			Persistent bacteremia at day 7 (n = 9)		
	n (%)	OR (95% CI)	aOR (95% CI)	n (%)	OR (95% CI)	aOR (95% CI)
Age (years)	-	0.96 (0.82–1.13)	NA	-	1.067 (0.95–1.2)	NA
Hospital onset	7 (87.5)	3.06 (0.35–26.48)	NA	8 (88.9)	3.73 (0.44–31.8)	NA
Primary source of infection						
None	3 (37.5)	2.85 (0.60–13.57)	4.51 (0.71–28.73)	0 (0.0)	NA	NA
CVC infection	3 (37.5)	0.55 (0.12–2.48)	NA	5 (55.6)	1.18 (0.29–4.77)	NA
Pneumonia	1 (12.5)	1.83 (0.19–17.96)	NA	3 (33.3)	10.5 (1.73–63.91)	8.63 (1.25–56.62)
BJI & SSTI	0 (0.0)	NA	NA	0 (0.0)	NA	NA
SSI	1 (12.5)	3.14 (0.29–34.42)	NA	1 (11.1)	2.63 (0.24–28.36)	NA
IE	0 (0.0)	NA	NA	0 (0.0)	NA	NA
Severe presentation	1 (12.5)	0.68 (0.08–6.04)	NA	2 (22.2)	1.429(0.26–7.82)	NA
Vancomycin MIC = 2 (µg/mL)	1 (12.5)	1.83 (0.19–17.96)	NA	1 (11.1)	1.525(0.16–14.76)	NA
ST groups						
ST1 group ^a^	3 (37.5)	39.0 (3.40–444.06)	44.12 (3.46–562.19)	2 (22.2)	17.71 (1.42–221.15)	14.37 (0.96–216.06)
ST5 group ^b^	0 (0.0)	NA	NA	1 (11.1)	0.48 (0.06–4.20)	NA
ST72 group ^c^	5 (62.5)	0.78 (0.17–3.56)	NA	5 (55.6)	0.54 (0.13–2.23)	NA

Abbreviations: OR, odds ratio; aOR, adjusted odds ratio; CI, confidence interval; CVC, central venous catheter; BJI, bone and joint infection; SSTI, skin and soft tissue infection; IE, infective endocarditis; MIC, minimal inhibitory concentration; ST sequence type; NA, not applicable; SLV, single locus variant

Notes: ^a^ ST1 (n = 3) and its SLVs, including novel SLV (n = 1)

^b^ ST5 (n = 9) and its SLVs including ST632(n = 1), novel SLV(n = 4).

^c^ ST72 (n = 47) and its SLVs including ST2084(n = 2), novel SLV(n = 1).

Considering microbiological outcomes, within the ST1 group, 50% (2/4) of cases exhibited persistent bacteremia at day 7, while the corresponding figures for the ST5 and ST72 groups were 7.1% (1/14) and 10% (5/50), respectively. In addition, MRSA bacteremic pneumonia was associated with persistent bacteremia at day 7 (aOR, 8.63; 95% CI, 1.25–56.62). Although univariate analysis indicated an association between the ST1 group and persistent bacteremia at day 7 (OR, 17.71; 95% CI, 1.42–221.15), after adjusting for bacteremic pneumonia and ST1 group, the ST1 group itself did not remain a significant risk factor for persistent bacteremia at day 7 (aOR, 14.37; 95% CI, 0.96–216.06).

### Antimicrobial resistance rates

All MRSA isolates were susceptible to vancomycin with 88.3% (68/75) having a vancomycin MIC of 1.0 *μg*/mL (Additional file 1: Table S1). The MDR rate was 38.8% (26/77), and the clindamycin resistance rate was 32.5% (25/77), including 20.8% (16/77) constitutive resistance and 31.0% (9/29) inducible resistance. No statistically significant differences in resistance rates were observed between the two periods for each antibiotic.

In comparison to the ST5 and ST1 groups, the MRSA ST72 group demonstrated lower overall antibiotic resistance and MDR rates among the three different ST groups (Additional file 1: Table S2). The clindamycin resistance rate within the ST5 group was 100%, while it was 12% (6/50) within the ST72 group. Among the five MRSA isolates with a vancomycin MIC of 2 *μg*/mL, three were from the ST72 group, and two were from the ST5 group.

## Discussion

This study focused on pediatric MRSA bacteremia demonstrates that ST72-SCC*mec* type IV MRSA has been the predominant strain between 2016 and 2021, replacing the ST5-SCC*mec* type II strain in hospital-onset infections among Korean children. Furthermore, the ST1 group was identified as an independent risk factor for 30-day all-cause mortality.

While MRSA rates in SAB have been decreasing in Europe and the United States since the 2000s [2, 19, 20], and a similar trend has been observed in adults in South Korea [21, 22], the MRSA rate among SAB cases in Korean children remained high, ranging from 51.9 to 76.1% between 2002 and 2016 [5]. However, during the study period, the incidence of pediatric MRSA bacteremia decreased by 23% annually, with MRSA accounting for 47.5% of SAB cases. Although the exact reason for this decreasing trend is unclear, the hospital`s efforts to strengthen infection prevention measures, including active MRSA surveillance, targeted precautions, and bundle approaches for CLABSI or ventilator-associated pneumonia, may have played a role. Notably, HO infections constituted approximately 70% of MRSA bacteremia cases, highlighting the significance of hospital-based infection control strategies.

Although understanding the molecular epidemiology of MRSA is crucial for controlling MRSA transmission in healthcare settings [6], there is limited pediatric data available. In the past, MRSA genotypes were distinguishable between community and hospital settings, but this differentiation has become challenging since the 1990s [4]. In our study, the predominant genotype was ST72-MRSA-SCCmec type IV, which was initially associated with the community but has now become prevalent in hospital settings, especially in the PICU. This observation aligns with the finding that ST72 has become endemic within the Korean pediatric population and exhibits effective spread among children in hospital settings [8, 23].

The study revealed that ST5, previously recognized as a pandemic hospital genotype, declined significantly during the study period. This finding is consistent with previous research in Korean adult populations, indicating a decrease in the prevalence of ST5 since the mid-2000s [7, 22]. This reduction could be related to infection control measures targeting central venous catheters, given that ST5 has been associated with *S. aureus* central-line related infection [22]. However, our study found that ST5 was being replaced by ST72 in pediatric MRSA CLABSI without significantly changing the overall incidence of CLABSI.

The ST1 (USA 400) emerged as the third most common genotype, with its prevalence remaining relatively constant throughout the study period, despite being infrequently detected in previous pediatric MRSA studies in Korea [8, 23]. ST1 has shown higher lethality in a rabbit model of *S. aureus* endocarditis [24], and lower virulence than ST8 in a rat model of MRSA pneumonia [25]. However, its impact on the clinical significance of human MRSA infections has rarely been reported. Our study revealed that the ST1 group was independently associated with higher mortality compared to the ST5 and ST72 groups. While an adult MRSA bacteremia study found that ST72 was independently associated with lower mortality than ST5 [26], our study did not reveal that ST72 or ST5 increased mortality with statistical significance. Overall, these findings underscore the importance of considering the specific ST of MRSA when assessing the clinical significance and mortality risk of MRSA infections. Different STs may possess varying virulence factors and impact patient outcomes differently. Further research to explore virulence factors according to ST will be necessary to validate the clinical significance of ST.

The ST72 group of MRSA strains exhibited relatively lower MDR and higher susceptibility to non-*β*-lactam antibiotics such as clindamycin, which can be considered as an option for empirical treatment in the outpatient setting. Our study revealed that all cases of BJIs and SSTIs occurred in the ST72 group, aligning with findings from a previous Korean pediatric study that identified ST72 as the most common ST in children with SSTI/BJI [27]. Considering the presence of inducible clindamycin resistance in a notable portion of ST72-MRSA isolates, performing a D-test is recommended to prevent treatment failure in MRSA infections like BJIs and SSTIs, which are frequently encountered in the community settings among Korean children.

Antibiotic resistance varied among genotypes; ST5, carrying SCCmec type IV, generally exhibited higher susceptibility to non-*β*-lactam antibiotics due to the absence of resistance-conferring genes compared to SCC*mec* types I and II [4]. Moreover, antibiotic resistance patterns could be influenced by spa types or regional differences [22, 28], and antibiotic pressure in hospital settings might contribute to the emergence of multi-drug resistance [29, 30]. In addition, certain genotypes may evolve easily, leading to the acquisition of resistance to specific antibiotics. For istance, ST5 showed increased resistance to chlorhexidine after the implementation of chlorhexidine bathing in hospital settings in Korea [30]. Fortunately, our study did not observe an increase in antibiotic resistance among ST72 during the study period. However, given the increasing prevalence of ST72 in hospital settings, it remains crucial to monitor any potential alterations in antibiotic resistance within this genotype.

There are several limitations to this study. Firstly, the findings cannot be extrapolated to other pediatric populations, including general pediatric population in South Korea, different hospitals, or different geographical areas. This is because the study was conducted solely at a single referral tertiary hospital, which serves a pediatric patient population with oncologic, hematopoietic stem cell transplantation, and solid organ transplantation backgrounds. Additionally, our study specifically focused on MRSA isolates obtained from pediatric patients with bacteremia, which may make its applicability more relevant to severe MRSA infections, such as complicated *S. aureus* bacteremia associated with underlying comorbidities. Secondly, the number of isolates analyzed may have been insufficient to achieve statistical significance when identifying risk factors for outcomes. However, the assessment of clinical impacts associated with specific STs in the context of pediatric patients in Korea, where ST72 predominates, is a rare endeavor. While our findings revealed that the ST72 group was not a risk factor for mortality or persistent bacteremia, consistent with previous adult studies [26], the ST1 emerged as an independent risk factor for 30-day all-cause mortality. Further research is warranted to delve into the factors contributing to clinical severity or virulence within ST1-MRSA strains. Large-scale studies might provide more robust statistical power to identify risk factors for outcomes. Thirdly, strain-specific virulence genes of MRSA isolates that could play pivotal roles in virulence were not identified in this study. Additionally, we did not establish associations between specific STs and clinical outcomes in relation to virulence factors. Therefore, further studies involving comprehensive whole-genome studies are necessary to validate the roles of virulence factors and their implications in clinical contexts.

## Conclusion

The ST72-MRSA strain was the most frequently identified strain over a 6-year period, from 2016 to 2021, among Korean children. ST1 was an independent risk factor for 30-day all-cause mortality in pediatric patients with MRSA bacteremia. Continous surveillance and monitoring of MRSA are important to gain a deeper understanding of the evolving epidemiology of MRSA and to inform effective strategies for prevention and treatment.

### Electronic supplementary material

Below is the link to the electronic supplementary material.

Supplementary Material 1

## Data Availability

All data generated or analysed during this study are included in this published article and its additional information files.
