# Supplementary material for: The molecular epidemiology and clinical implication of methicillin-resistant Staphylococcus aureus (MRSA) sequence types in pediatric bacteremia: a restrospective observational study, 2016–2021
Source: BMC Infect Dis. 2024 Feb 24;24:259. doi: 10.1186/s12879-023-08914-5 (PMC10894466; doi:10.1186/s12879-023-08914-5)
Supplement: Supplementary file 1 — Supplementary Material 1 [file 12879_2023_8914_MOESM1_ESM.docx]

**Additional file 1**

**Table S1. Antimicrobial resistance rate during the two study periods**

|  |  | **All STs (total n=77)** | | | **ST72 group (total n=50)** | | |
| --- | --- | --- | --- | --- | --- | --- | --- |
| **Antibiotic** | **Total**  **(2016-2021)**  **(n=77)** | **Period 1**  **(2016–2018)**  **(n=52)** | **Period 2**  **(2019–2021)**  **(n=25)** | ***P*-value** | **Period 1**  **(2016–2018)**  **(n=31)** | **Period 2**  **(2019–2021)**  **(n=19)** | ***P*-value** |
| Multidrug resistant ^a^ | 26 (38.8) | 18 (34.6) | 8 (32.0) | 0.820 | 2 (6.5) | 4 (21.1) | 0.184 |
| Erythromycin | 29 (37.7) | 19 (36.5) | 9 (36.0) | 0.835 | 4 (12.9) | 4 (21.1) | 0.459 |
| Ciprofloxacin | 23 (29.9) | 16 (30.8) | 7 (28.0) | 0.804 | 2 (6.5) | 2 (10.5) | 0.629 |
| TMP/SMX | 4 (5.2) | 2 (3.8) | 2 (8.0) | 0.592 | 0 (0.0) | 1 (5.3) | 0.380 |
| Tetracycline | 19 (24.7) | 16 (30.8) | 3 (12.0) | 0.074 | 4 (12.9) | 0 (0.0) | 0.284 |
| Rifampin | 3 (3.9) | 2 (3.8) | 1 (4.0) | >0.99 | 0 (0.0) | 1 (5.3) | 0.380 |
| Fusidic acid | 14 (18.2) | 10 (19.2) | 4 (16.0) | >0.99 | 0 (0.0) | 1 (5.3) | 0.380 |
| Mupirocin | 13 (16.9) | 8 (15.4) | 5 (20.0) | 0.747 | 4 (12.9) | 3 (15.8) | >0.99 |
| Gentamycin | 30 (39.0) | 21 (40.4) | 9 (36.0) | 0.835 | 5 (16.1) | 4 (21.1) | 0.715 |
| Clindamycin ^b^ | 25 (32.5) | 19 (36.5) | 6 (24.0) | 0.271 | 3 (9.7) | 3 (15.8) | 0.661 |
| Clindamycin iR | 9 (31.0) ^c^ | 6 (30.0) ^c^ | 3 (33.3) ^c^ | >0.99 | 2 (50.0) ^c^ | 3 (75.0) ^c^ | >0.99 |
| Vancomycin |  |  |  |  |  |  |  |
| MIC of 2 μg/mL | 6 (7.8) | 3 (5.8) | 3 (12.0) | 0.383 | 1 (3.2) | 2 (10.5) | 0.549 |
| MIC of 1 μg/mL | 66 (85.7) | 47 (90.4) | 19 (76.0) | 0.161 | 29 (93.5) | 14 (73.7) | 0.089 |
| MIC of ≤ 0.5 μg/mL | 5 (6.5) | 2 (3.8) | 3 (12.0) | 0.322 | 1 (3.2) | 3 (15.8) | 0.147 |

Data are presented as numbers (%) of patients.

Abbreviation: TMP/SMX, trimethoprim-sulfamethoxazole; iR, erythromycin-inducible resistance

Notes: ^a^ Multidrug-resistant, resistant to three or more different classes of non-*ß*-lactam antibiotics.

^b^ Clindamycin resistance encompasses both types of resistance, inducible and constitutive.

^c^ Percentage of erythromycin-inducible clindamycin resistant isolates among total erythromycin-resistant isolates.

**Table S2.** Antibiotics resistant rate and clinical characteristics of MRSA blood isolates according to the ST groups

| **Characteristic** | **ST72 group ^a^**  **(N=50)** | **ST5 group ^b^**  **(N=14)** | **ST1 group ^c^**  **(N=4)** | ***P*-value** |
| --- | --- | --- | --- | --- |
| Resistance rate |  |  |  |  |
| Multidrug resistant ^d^ | 6 (12.0) | 14 (100.0) | 2 (50.0) | <0.001 |
| Erythromycin | 8 (16.0) | 14 (100.0) | 2 (50.0) | <0.001 |
| Ciprofloxacin | 4 (8.0) | 14 (100.0) | 0 (0.0) | <0.001 |
| TMP/SMX | 1 (2.0) | 2 (14.3) | 0 (0.0) | 0.169 |
| Tetracycline | 4 (8.0) | 11 (78.6) | 2 (50.0) | <0.001 |
| Rifampin | 1 (2.0) | 1 (7.1) | 0 (0.0) | 0.462 |
| Fusidic acid | 1 (2.0) | 11 (78.6) | 0 (0.0) | <0.001 |
| Mupirocin | 7 (14.0) | 2 (14.3) | 2 (50.0) | 0.154 |
| Gentamycin | 9 (18.0) | 12 (85.7) | 4 (100.0) | <0.001 |
| Clindamycin ^e^ | 6 (12.0) | 14 (100) | 2 (50.0) | <0.001 |
| Clindamycin iR | 5 (62.5) ^f^ | 2 (14.3) ^f^ | 2 (100.0) ^f^ | 0.013 |
| Vancomycin MIC |  |  |  |  |
| 2 ㎍/mL | 3 (6.0) | 2 (14.3) | 0 (0.0) | 0.487 |
| 1 ㎍/mL | 43 (86.0) | 12 (85.7) | 4 (100.0) | >0.99 |
| ≤ 0.5 ㎍/mL | 4 (8.0) | 0 (0.0) | 0 (0.0) | 0.663 |
| Primary source of infection |  |  |  |  |
| CVC-related infection | 25 (50.0) | 8 (57.1) | 1 (25.0) | 0.893 |
| BJI&SSTI | 9 (18.0) | 0 (0.0) | 0 (0.0) | 0.155 |
| Pneumonia | 4 (8.0) | 1 (7.1) | 0 (0.0) | 0.498 |
| SSI | 3 (6.0) | 1 (7.1) | 0 (0.0) | 0.864 |
| Infective endocarditis | 2 (4.0) | 1 (7.1) | 2 (50.0) | 0.798 |
| Primary bacteremia | 7 (14.0) | 3 (21.4) | 1 (25.0) | 0.709 |

Data are presented as number (%) of patients. *P*-values were obtained using Fisher’s exact tests for the three groups (ST72, ST5, and ST1 groups).

Abbreviations: iR, erythromycin-inducible resistance; TMP/SMX, trimethoprim–sulfamethoxazole; CVC, central venous catheter;
BJI, bone and joint infection; SSTI, skin and soft tissue infection; SSI, surgical site infection; SLV, single locus variant.

Notes: ^a^ ST72 (n= 47) and its SLVs, including ST2084 (n=2), novel SLV (n=1).

^b^ ST5 (n= 9) and its SLVs, including ST632 (n=1), novel SLV (n=4).

^c^ ST1 (n=3) and its SLVs, including novel SLV (n=1).

^d^ Multidrug-resistant, resistant to three or more different classes of non-*ß*-lactam antibiotics.

^e^ Clindamycin resistance encompasses both types of resistance, inducible and constitutive.

^f^ Percentage of erythromycin-inducible clindamycin resistant isolates among total erythromycin-resistant isolates.
